# Genome Sequences of Lactococcus garvieae and Lactococcus petauri Strains Isolated from Traditional Montenegrin Brine Cheeses

**DOI:** 10.1128/MRA.00546-21

**Published:** 2021-08-05

**Authors:** Aleksandra Martinovic, Adriana Cabal, Andjela Nisic, Jasmin Sucher, Anna Stöger, Franz Allerberger, Werner Ruppitsch

**Affiliations:** a Centre of Excellence for Digitalization of Microbial Food Safety Risk Assessment and Quality Parameters for Accurate Food Authenticity Certification (FoodHub), University of Donja Gorica, Podgorica, Montenegro; b Institute of Medical Microbiology and Hygiene, Austrian Agency for Health and Food Safety, Vienna, Austria; c Department of Biotechnology, University of Natural Resources and Life Sciences, Vienna, Austria; University of Rochester School of Medicine and Dentistry

## Abstract

Lactococcus garvieae and Lactococcus petauri cause lactococcosis in fish. Both species have also been isolated from various food products and are considered emerging zoonotic pathogens. Here, we report the genomes of L. garvieae INF126 and *L. petauri* INF110, obtained from traditional Montenegrin brine cheeses.

## ANNOUNCEMENT

Lactococcus garvieae and Lactococcus petauri are members of the lactic acid bacteria (LAB) group. L. garvieae was first described as Streptococcus garvieae in 1983 (1) and subsequently separated into subgroups A and B (2). Genomic analysis reassigned L. garvieae subgroup A strains to the recently described species *L. petauri* (3). Consequently, both species can be considered the etiological agents of lactococcosis in fish (4, 5) and emerging opportunistic zoonotic pathogens (6–8). The isolation of both species from a variety of food (9–11) also implicates a contribution to the quality and typicity of various traditional food products (10). Comparison with other sequenced strains may provide new information on their safety (12), adaptation to diverse environments, and importance for traditional food products (10).

Enrichment and isolation of bacterial isolates from traditional Montenegrin white brine cheeses was performed using M17 and de Man, Rogosa, and Sharpe (MRS) broth (both from HiMedia, India) according to the method previously described (13). Colonies morphologically suspected to be LAB were subcultured on MRS agar for species identification by matrix-assisted laser desorption ionization–time of flight mass spectrometry (MALDI-TOF MS) (Microflex LT/SH, MBT Compass IVD 4.2; Bruker, Billerica, MA, USA) and whole-genome sequencing (WGS).

For WGS, genomic DNA was obtained from overnight cultures grown on MRS agar at 37°C using the MagAttract high-molecular-weight (HMW) DNA kit (Qiagen, Hilden, Germany). Libraries were prepared using Nextera XT (Illumina, Inc., San Diego, CA, USA), and 2 × 300-bp sequencing was performed on a MiSeq instrument (Illumina, Inc.) as previously described (14).

Default parameters were used for all software unless otherwise specified. FastQC v0.11.9. was used to control raw read quality, Trimmomatic v0.36 (15) was used to remove adapter sequences and to trim the last 10 bp of each sequence and sequences with quality scores of <20, and SPAdes v3.15.2 (16) was used for read assembly. Contigs were filtered for a minimum coverage of 5-fold and a minimum length of 200 bp using SeqSphere+ software v7.5.2 (Ridom GmbH, Würzburg, Germany).

WGS of *L. petauri* INF110 and L. garvieae INF126 generated 1,839,606 and 1,035,909 reads, respectively. Assemblies resulted in 172 and 149 contigs with a mean coverage of 55- and 41-fold and a GC content of 37.9% and 38.8%, respectively. The NCBI Prokaryotic Genome Automatic Annotation Pipeline (17) identified 2,256 and 2,383 genes, 2,197 and 2,244 coding sequences, 163 and 70 pseudogenes, and 59 and 69 RNA genes, respectively (Table 1).

**TABLE 1:** Characteristics and accession numbers of genomes of L. garvieae isolates from Montenegrin brine cheese^*a*^

Strain	Species (dDDH and rMLST)	Genome size (Mb)	GC content (%)	No. of reads	Total no. of genes	CDS	No. of RNA genes	Avg coverage (×)	No. of contigs	Contig *N*_50_ (bp)	AMR genes	MGE	Plasmids	Bacteriocin gene	GenBank accession no.	SRA accession no.
INF110	*L. petauri*	2.2	37.9	1,839,606	2,256	2,197	59	55	172	50,638	*mdt*(A), *clpI*	ISLll1, ISS1N, ISS1X, IS-LL6	pVF21 (PLACNETw: 11,324 bp/21,739 bp; REL, 98.333%; RIP, 98.695%), pGL5 (PLACNETw: 34.207 bp/68,798 bp; no REL/RIP sequences; BLAST: 83.61–97.92% identity/19% coverage)	*garQ*	JAGYXE000000000	SRR14581598
INF126	L. garvieae	2.3	38.8	1,035,909	2,383	2,244	59	41	149	217,709	*lsa*(D), *clpI*	IS1068, ISS1N, ISTeha2	rep33 (ResFinder), pClS8 (PLACNETw: 40,920 bp/80,592 bp; REL, 96.667%; RIP, 78.553%), pGL5 (PLACNETw: 18,661 bp/68,798 bp; REL, 94.667%; RIP, 83.069%; BLAST: 96.33–99.27% identity/9% coverage)	*garQ*	JAGYXD000000000	SRR14581597

a Isolates were identified as nonpathogenic (CGE-PathoFinder). AMR, antimicrobial resistance; MGE, mobile genetic elements; REL, relaxases; RIP, replication initiation proteins; rMLST, ribosomal multilocus sequence typing; CDS, coding DNA sequences.

MALDI-TOF identified both isolates as L. garvieae. Digital DNA-DNA hybridization (dDDH) (18) identified INF110 as *L. petauri* with similarities of 82.3% to *L. petauri* 159469^T^ and 50.7% to L. garvieae ATCC 49156^T^. INF126 was identified as L. garvieae with 80.2% similarity to L. garvieae ATCC 49156^T^ and 54.8% to *L. petauri* 159469^T^. The average nucleotide identity (ANI) (19) between INF110/INF126 and *L. petauri* 159469^T^ and L. garvieae ATCC 49156^T^ was 97.21% and 92.80% and 92.32% and 97.56%, respectively. A gene-by-gene comparison with an *ad hoc* core genome scheme comprising 1,268 targets using SeqSphere+ with default settings and strain ATCC 49156 as a reference showed 1,177 allelic differences between INF110 and INF126 and no similarities to other strains deposited in GenBank. For safety evaluation of pathogenicity and antimicrobial resistance, plasmids and mobile genetic elements were determined through the tools available from the Center for Genomic Epidemiology (http://www.genomicepidemiology.org/) and PLACNETw (20), respectively (Table 1).

### Data availability.

The whole-genome shotgun (WGS) project has been deposited in DDBJ/ENA/GenBank under the BioProject PRJNA727069 with the accession no. JAGYXE000000000 (INF110) and JAGYXD000000000 (INF126). The versions described in this paper are the first versions, JAGYXE010000000 and JAGYXD010000000. The raw sequence reads have been deposited in the Sequence Read Archive (SRA) under accession no. SRR14581598 (INF110) and SRR14581597 (INF126).
